# Suggestion of new possibilities in approaching individual variability in appetite through constitutional typology: a pilot study

**DOI:** 10.1186/1472-6882-12-122

**Published:** 2012-08-13

**Authors:** Junhee Lee, Jiwon Lee, Hyunshang Shin, Ki-Suk Kim, Euiju Lee, Byunghee Koh, Hyeung-Jin Jang

**Affiliations:** 1 Department of Sasang Constitutional Medicine, College of Oriental Medicine, Kyung Hee University; 2 Department of Biochemistry, College of Oriental Medicine, Kyung Hee University, #1 Heogi-dong, Dongdaemun-gu, Seoul 130-701, Republic of Korea

**Keywords:** Gut hormone, Appetite, Eating behavior, Sasang constitutional typology

## Abstract

**Background:**

Appetite is intricately connected to eating behaviors and shows a high individual variability. In an attempt to approach the problem of gut hormone profiles, appetite, and eating behaviors at the individual level, we have adopted a constitutional typing system widely used in traditional East-Asian medicine, the Sasang constitutional typology, in order to determine the individual variations in appetite, eating behavior, and weight change.

**Methods:**

This pilot study was designed to investigate the variability of appetite among individuals by tracking the gut hormone patterns across different constitutional types. Pre- and post-prandial concentrations of anorectic (peptide YY (PYY), glucagon-like peptide 1 (GLP-1)) and orexigenic (active ghrelin) gut hormones were measured in healthy, normal-weight (18.5 kg/m^2^ ≤BMI <23 kg/m^2^) male subjects aged 20–35 (Soyang (SY) (n = 9), Taeeum (TE) (n = 9), and Soeum (SE) (n = 10) constitutional types).

**Results:**

Significant differences were found only in the PYY concentrations across the three groups (p = 0.031). The PYY concentration peaked at 30-min post-prandial in the SE group and was significantly higher compared to the other two groups (p = 0.004). The GLP-1 concentration peaked at 15-min post-prandial in the SE group (not significant). The ghrelin levels at 30-min pre-prandial were relatively lower in the TE group compared to the other groups (not significant).

**Conclusions:**

In conclusion, although with weak statistical power, meaningful gut hormone patterns specific to each constitutional type were discovered in this pilot study, which could offer a new method of approaching the problem of appetite and eating behavior from the angle of individual variability in appetite.

## Background

The gastrointestinal tract is the largest endocrine organ that secretes the gut hormones that play important roles in energy metabolism and weight control. Several gut hormones have been discovered so far, of which many are closely associated with appetite and systemic metabolism [1,2]. Appetite is also closely related to eating behavior and shows a high variability among different individuals [3]. Many studies have been conducted based on this close association between gut hormones, appetite, and metabolism. Some studies on gut hormone profiling and appetite have compared the healthy population versus unhealthy population (e.g. populations with obesity or anorexia nervosa) [1], and some have investigated the genetic associations of appetite and gut hormones in the healthy population [4-6]. However, except these population-based studies, no study has yet explored the appetite patterns at the individual level through gut hormone profiling.

In clinical settings, case-specific traits of individual patient can be more meaningful for predicting his or her physiologic and pathologic status than average features of the whole population. For this reason, healthcare providers often rely on their own clinical experiences rather than clinical standards to infer pathological patterns of patients and to make medical decisions. Especially concerning medical subjects of complex nature, such as appetite and eating behavior, many agree that an approach from a different angle factoring in the individually diversified constitutional make-up of an individual would be more efficient. In one such attempt, we have explored the individualized patterns of appetite and eating behaviors through a widely used constitution typing system, the Sasang constitutional typology.

The Sasang constitutional medicine (SCM) is a component of traditional Korean medicine that studies the typing of individuals into four constitutional types Taeyangin (TY type), Soyangin (SY type), Taeeumin (TE type) and Soeumin (SE type) based on their physical and psychological attributes (Table 1) [7,8]. The physiology, pathology, and therapeutics specific to each constitutional type have been explored extensively in many studies [9]. Several recent studies also report different prevalence rates of chronic diseases across different Sasang constitutional types, in obesity [10,11], insulin resistance [12], diabetes mellitus [13], hypertension [14], metabolic syndrome [15], and cerebrovascular diseases [16,17], providing scientific evidence of the well-recognized patterns already witnessed by clinicians.

**Table 1:** General characteristics of the four Sasang constitutional types

	**TY type**	**SY type**	**TE type**	**SE type**
**Hyperactive organ system**	Lung*	Spleen*	Liver*	Kidney*
**Hypoactive organ system**	Liver*	Kidney*	Lung*	Spleen*
**Insidious greed and deviations**	Indiscipline, Ignobility	Pretentiousness, Frivolity	Acquisitiveness, Avarice	Stealthiness, Prevarication
**Personality**	Communicative, decisive	Forceful, strong-willed	Consistent, reliable	Prudent, composed
**Sign of health**	Plentiful urination	Brisk perspiration	Easy defecation	Comfortable digestion
**Sign of illness**	Restricted urination	Obstructed perspiration	Hindered defecation	Strained digestion

* These represent the functional and structural conceptualization of organs specific to the Sasang constitutional typology that are completely different from their namesake organs in Western medicine. In Sasang constitutional medicine, the variegated functions of the human body is summarized into the interactions of the four organ systems that represent the following roles in the body: the Lung system is involved in the process of dispersing the energy-fluid material, the Liver system in concentrating the energy-fluid material, the Spleen system in the containing of the water-food material, and the Kidney system in discharging of the water-food material.

[adapted from Kim et al.[8]**].**

These studies show that the Sasang constitutional typology could be a useful tool in approaching the patient from the patient-specific or constitution-based angle, and indeed many practitioners had been successfully using the Sasang typology in educating patients and predicting pathological progressions. In particular, SCM suggests that different Sasang constitutional types exhibit different clinical traits in appetite, eating behavior, and weight change [7,18].

This pilot study was designed to investigate for the first time the variability of appetite among individuals across different Sasang constitutional types by tracking the pre- and post-prandial concentrations of the anorectic gut hormones (peptide YY (PYY), and glucagon-like peptide 1 (GLP-1)) and the orexigenic gut hormone (active ghrelin) in healthy individuals.

## Methods

### Subjects

We recruited male subjects aged 20–35, who were in normal weight range (18.5 kg/m^2^ ≤BMI <23.0 kg/m^2^) and confirmed on their Sasang constitutional types (SY, TE, or SE). The inclusion and exclusion criteria are presented in Table 2. This study was conducted according to the guidelines laid down in the Declaration of Helsinki and all procedures involving human subjects were approved by the Institutional Review Board at the Hospital of Korean Medicine of Kyung Hee University. Written informed consent was obtained from all subjects with full understanding of the purpose and procedure of the study.

**Table 2:** Inclusion and exclusion criteria

**Criteria**
Inclusion - Male
- Aged 20-35
- 18.5 kg/m^2^ ≤Body mass index (BMI) <23 kg/m^2^
- Sasang-constitutionally typed as either the SY, the TE, or the SE type
Exclusion- History of impaired fasting glucose or diabetes mellitus (past history of diabetes or fasting blood glucose at screening ≥100 mg/dl)
- History of liver disease (hepatitis, hepatic cirrhosis) or hepatic dysfunction (AST or ALT at screening ≥40 U/L)
- History of renal dysfunction (creatinine at screening ≥1.2 mg/dl)
- History of heart disease (heart failure, angina pectoris, myocardial infarction, arrhythmia)
- History of malignant tumor
- Possibility of anorexia nervosa, bulimia nervosa, or other similar disorders (applicants satisfying the DSM-IV criteria for eating disorder and those who scored ≥88 in the Bulimia Test Revised or ≥21 in the Korean Version of Eating Attitude Test-26 were eliminated)
- Having digestive disorders that can interfere with normal absorption of standard diet (gastritis, gastric ulcer, duodenitis, duodenal ulcer, etc.)
- Smoking during the recent 3 months
- Alcohol consumption 3 or more times a week during the recent 3 months
- Weight change greater than ±2 kg during the recent 3 months
- Anomalous eating pattern (skipping breakfast or having breakfast before 6AM or after 10AM)
- Medicated during the recent month for therapeutic or prophylactic purposes
- Participating in another clinical trial

All applicants were surveyed beforehand on their demographic characteristics, past medical history, and lifestyle patterns. The screening also included body size measurement, blood tests, questionnaires (Bulimia Test Revised (BULIT-R) [19], Korean Version of Eating Attitude Test-26 (KEAT-26) [20], Gastrointestinal Symptom Rating Scale (GSRS) [21], and Dutch Eating Behavior Questionnaire (DEBQ) [22,23]), and Sasang constitutional typing. GSRS and DEBQ were each used to assess the overall gastrointestinal symptoms and the eating behaviors of the participants. After non-eligible applicants were screened out, the remaining participants were assigned identification codes, and all personal and identifying information was blinded from the researchers until the end of trial.

Of the initial 57 applicants, 7 were eliminated due to high BMI and 1 for showing anomalous eating patterns. Sasang constitutional typing was performed on the remaining 49, and 14 whose constitutional typing by two specialists did not match each other were dropped. After further eliminating 3 who showed abnormal blood test results and 1 who withdrew initial consent, 31 participants remained in the final subject group (SY (n = 10), TE (n = 11), and SE (n = 10) types).

### Sasang constitutional typing

Two SCM specialists examined the participants and determined their constitutional types based on their physical, mental, physiological, and pathological attributes. The SCM specialists were qualified traditional Korean medical doctors licensed by the Korean government, with six years of traditional Korean medicine training, minimum three years of training in SCM, and nine years of further clinical experience. The two specialists were mutually blinded, and used structured and pre-determined questionnaire to improve objectiveness of decision making. The constitutional type was confirmed only when both made concordant decisions. Of the 49 who were examined by the specialists, 35 were typed identically, yielding a diagnostic synchronicity of 71.4%.

### Experimental procedure

The subjects were given information on the study protocol 3 days beforehand and were forbidden to exercise, drink alcohol, overeat, or fast during the 48 hours preceding the trial. Each was distributed a standard meal (800 kcal liquid diet (Medifood Standard, Korea Medical Foods Co., Ltd); 58% carbohydrate, 15% protein, 27% lipid) to be ingested between 6PM and 7PM on the day before trial to be followed by fasting from 8PM onward.

On the day of trial, participants arrived at 7AM, shortly after which the height, weight, and body composition were measured, and trained nurses inserted intravenous catheters for blood sampling. Samples for basic blood test (fasting blood glucose, insulin, triglyceride, total cholesterol, high density lipoprotein cholesterol, and low density lipoprotein cholesterol) were taken simultaneously. After 60 minutes of rest, they were given 10 minutes to ingest the 800 kcal standard diet. Samples were taken at six time-points – 30-minutes pre-prandial, immediately before ingestion, and 15, 30, 60, 120-minutes post-prandial. The participants were asked to remain seated throughout the experiment (allowing only light activities such as writing, reading, watching video, talking, using the toilet, etc.) and were prohibited from exercising or sleeping.

### Sample processing

Blood samples were collected in ice-cooled Vacutainer EDTA-plasma tubes. Immediately (< 30 seconds) after sampling, DPP-IV inhibitor (Linco Cat # DPP4) was added in accordance to the manufacturer’s instructions (10 μl DPP-IV inhibitor per milliliter of blood), and the tube was inverted to mix and stored in ice bath. Immediately afterwards, sampling tubes were centrifuged at 1000 xg for 10 minutes and stored at −70°C to be analyzed afterwards in ELISA.

The ELISA method (using ELISA KIT, 96-well plate (Millipore, Missouri, USA)) was used to assay the plasma concentrations of GLP-1, PYY, and ghrelin. The assay buffer was used as negative control and the provided standard sample as positive control. Two kinds of quality control solutions were provided in each kit. All controls and samples were added to the well in pairs. All procedures were correctly performed according to the manufacturer’s instructions. Fluoroskan Ascent FL (MTX Labsystems, VA, USA) was used to obtain raw data. Missing data was found across three or more time-points in 3 of 31 participants’ samples (1 in the SY group and 2 in the TE group) and therefore omitted in the final analysis.

### Statistical analysis

All values are presented as median (minimum, maximum). Kruskal-Wallis test was performed for a three-group analysis for variables with single assessment, and multiple comparison using the Mann–Whitney U test was performed (employing two-tailed Bonferroni-corrected p-value of <0.017 to minimize type I error). Generalized estimating equation for repeated-measures was used to analyze the gut hormone levels to detect group-by-time interactions, group-by-BMI interactions and inter-group differences. The Kruskal-Wallis test and multiple comparisons using the Mann–Whitney U test were done similarly for the inter-group comparison of the appetite-regulating hormones at each time-point. All statistical analyses were performed using the PASW Statistics 18.0 for Windows (Chicago, IL).

## Results

### General characteristics

All subjects were within normal BMI range, but the SE type weighed significantly less than the SY and TE types (p = 0.002), the BMI being highest in the TE group and lowest in the SE group (p <0.001). Waist circumference, hip circumference, blood pressure, pulse rate, fasting glucose level, and lipid profiles were not significantly different across the three constitutional types.

Eating attitude (assessed by KEAT-26) was normal in all three groups, and bulimic tendency (assessed by BULIT-R) was also normal in all the groups, though significantly higher in the TE type compared to the SE type. When the eating behavior (assessed by DEBQ) was compared, restraint eating showed significant difference across all three constitutional types (p = 0.044) (though not significant in multiple comparison). When the overall gastrointestinal symptoms (using GSRS) were evaluated, none of the three constitutional types showed remarkable symptoms, and no significant inter-group difference was discovered (p = 0.677) (Table 3).

**Table 3:** Characteristics of participants in different Sasang constitutional groups

		**SY type**		**TE type**		**SE type**		**p-value**
		**(n = 9)**		**(n = 9)**		**(n = 10)**		
Age (year)		23.0	(20.0, 27.0)	22.0	(20.0, 24.0)	22.5	(20.0, 25.0)	0.975
Height (cm)		178.2	(165.1, 187.7)	178.5	(166.8, 182.6)	175.2	(163.8, 184.3)	0.414
Weight (kg)		71.4	(58.9, 76.4) ^a^	72.1	(63.5, 76.2) ^a^	61.2	(55.1, 67.7) ^b^	0.003
Body mass index (kg/m^2^)		21.6	(20.2, 23.0) ^b^	22.5	(21.9, 23.0) ^a^	20.0	(18.6, 21.7) ^c^	0.000
Waist circumference (cm)		77.5	(69.8, 85.0)	82.0	(74.8, 84.3)	76.5	(69.5, 79.0)	0.059
Hip circumference (cm)		95.5	(85.7, 100.0)	96.5	(92.5, 102.5)	93.4	(85.5, 99.0)	0.096
Pulse rate (/min)		78.0	(66.0, 84.0)	72.0	(66.0, 84.0)	72.0	(60.0, 84.0)	0.319
Systolic blood pressure (mmHg)		105.0	(85.0, 135.0)	117.5	(95.0, 135.0)	102.5	(95.0, 110.0)	0.257
Diastolic blood pressure (mmHg)		70.0	(60.0, 85.0)	70.0	(60.0, 85.0)	70.0	(50.0, 75.0)	0.683
Fasting glucose (mg/dL)		85.0	(77.0, 91.0)	89.0	(78.0, 101.0)	83.0	(79.0, 92.0)	0.354
Triglyceride (mg/dL)		66.0	(32.0, 95.0)	61.0	(31.0, 230.0)	57.5	(41.0, 136.0)	0.841
Total cholesterol (mg/dL)		147.0	(130.0, 180.0)	148.0	(102.0, 187.0)	148.0	(119.0, 174.0)	0.944
HDL cholesterol (mg/dL)		56.0	(42.0, 70.0)	57.0	(37.0, 79.0)	59.0	(50.0, 79.0)	0.758
LDL cholesterol (mg/dL)		89.0	(79.0, 111.0)	79.0	(58.0, 121.0)	83.5	(53.0, 116.0)	0.520
Insulin (µU/mL)		5.6	(3.7, 10.1)	7.1	(3.5, 18.1)	7.4	(4.4, 18.7)	0.494
HOMA-IR		1.1	(0.7, 2.1)	1.5	(0.7, 4.1)	1.4	(0.8, 4.2)	0.483
KEAT-26		3.0	(0.0, 13.0)	0.0	(0.0, 12.0)	3.5	(0.0, 10.0)	0.232
BULIT-R		47.0	(31.0, 87.0) ^ab^	45.0	(31.0, 54.0) ^a^	38.5	(31.0, 44.0) ^b^	0.037
DEBQ	Restraint	24.0	(17.0, 37.0)	25.0	(12.0, 34.0)	15.0	(11.0, 25.0)	0.044
	Emotional	19.0	(13.0, 50.0)	23.0	(13.0, 34.0)	13.5	(13.0, 18.0)	0.060
	External	32.0	(19.0, 45.0)	28.0	(25.0, 36.0)	25.0	(14.0, 37.0)	0.396
GSRS		3.0	(1.0, 6.0)	3.0	(0.0, 7.0)	2.5	(0.0, 9.0)	0.677

All values are presented as median (minimum, maximum).

^abc^ Values in the same row that are marked with different superscript alphabets are significantly different (p <0.017).

HOMA-IR, homeostasis model assessment of insulin resistance; KEAT-26, the Korean Version of Eating Attitude Test-26; BULIT-R, Bulimia Test Revised; DEBQ, Dutch Eating Behavior Questionnaire; GSRS, Gastrointestinal Symptom Rating Scale.

### Gut hormone profiling

**Figure 1:**
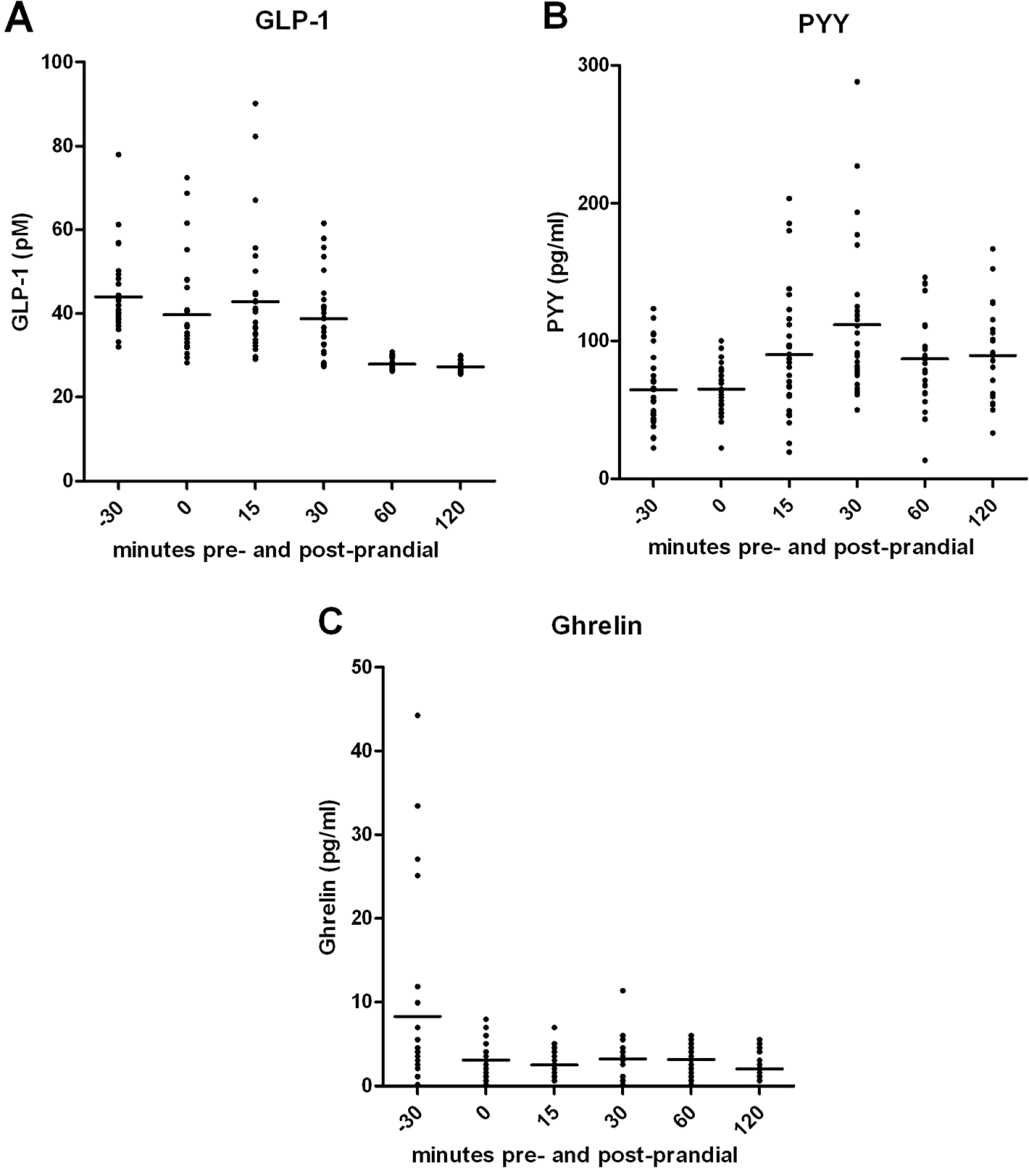
**Distributions of pre- and post-prandial gut hormone levels.** The horizontal lines in each figure are means.

The pre- and post-prandial gut hormone levels of all subjects were shown in Figure 1. The distributions of gut hormone levels, especially ghrelin at 30-minutes pre-prandial, were rather wide due to the small sample size.

**Figure 2:**
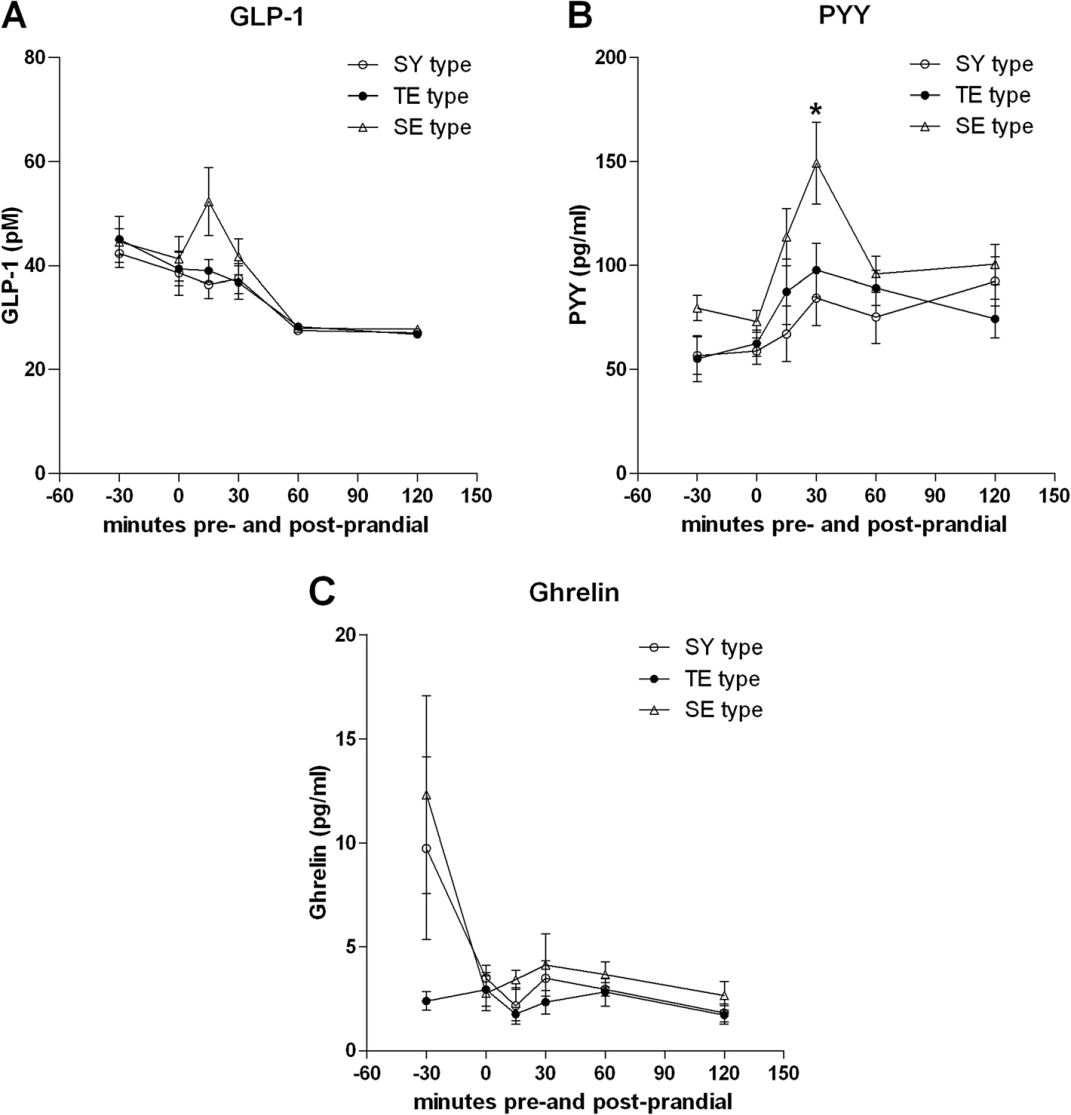
**Pre- and post-prandial plasma levels of gut hormones across different Sasang constitutional types.** Plotted in each figure are means and standard error of mean (SEM). **(A)** Glucagon-like peptide (GLP-1) **(B)** Peptide YY (PYY) **(C)** Ghrelin. Asterisk (*) denotes significant difference between different Sasang constitutional types at each time-point (p <0.017).

Time-group interaction was found in GLP-1, PYY, and ghrelin, but there were no group-by-BMI effects. The SE group showed a peak in the GLP-1 concentration at 15-minutes post-prandial, in contrast to its absence in the TE and SY curves. At 15-minutes post-prandial, the SE GLP-1 level was higher than that of the other two groups, but not with statistical significance (p = 0.096) (Figure 2-A).

The SE group displayed a marked peak in the PYY concentration at 30-minutes post-prandial, but this peak was absent in the TE and SY groups. At 30-minutes post-prandial, the PYY level in the SE group was significantly higher compared to the other two groups (p = 0.004) (Figure 2-B).

The ghrelin levels at 30-minutes pre-prandial in the SE and SY groups were relatively higher compared to that of the TE group. However, none of the time-points yielded significant inter-group differences (Figure 2-C).

## Discussion

When gut hormone profiles (GLP-1, PYY, and ghrelin) were analyzed in healthy, normal-weight, male subjects, gut hormone patterns were found to vary across different Sasang constitutional types.

The most important finding in this study is that the SE group displayed a peak in PYY levels 30 minutes after food intake, which was absent in the SY and TE groups. Furthermore, the PYY level at the 30-minutes post-prandial time-point was significantly higher in the SE group than in the other two groups. This presence of a marked post-prandial PYY peak found only in the SE group could be suggestive of strong PYY-mediated appetite suppression in the SE type as compared to the other constitutional types. PYY is an anorectic gut hormone related to satiety induction that increases in the bloodstream approximately 15 minutes following food intake, reaching peak levels at 1–2 hours and remaining elevated for several hours thereafter [24]. The SE constitutional type is known to display low appetite, small eating capacity, and greater dyspeptic tendencies, in contrast to the TE type who shows higher appetite, larger eating capacity, and greater risk for overweight and obesity. When these constitutional traits well-documented in literature are connected with the physiological role of PYY as a satiety factor, it could explain the accentuated post-prandial PYY peak in the SE group and the blunted post-prandial response in the TE group. Also, post-prandial PYY response is known to be blunted in obese persons [25], and the fact that the normal-weight TE group in our study shows this pattern could be interpreted as supporting the higher prevalence and risk for obesity found in the TE type in previous studies.

Other potentially meaningful gut hormone patterns were found in GLP-1, that could be interpreted in context of the known traits specific to each constitutional type. There was a peak in the GLP-1 curve in the SE type at 15-minutes post-prandial, although no significant difference was detected when compared with the SY or TE groups. GLP-1, an anorectic gut hormone [26], is released by food intake, upon which its circulating concentration increases biphasically within 10 minutes, peaking at 30 minutes, and remains elevated for several hours [27]. This pattern of response to food ingestion suggests that GLP-1 could play a role in meal termination and possibly in generating satiety and delaying gastric emptying [28]. Though not significant, it is interesting that only the SE group showed a post-prandial peak in GLP-1 in light of the known traits of the SE and TE constitutional types.

The TE group failed to display an elevation in ghrelin level prior to food ingestion and showed a relatively lower (though not significant) ghrelin concentration compared to the SE and SY groups at 30-minutes pre-prandial. In literature, the plasma level of ghrelin, the only gut hormone known to increase food intake, rises before the start of a meal and drops again shortly after meal initiation [29]. Studies show that individuals with high BMI actually display low circulating ghrelin, lack the typical ghrelin spikes throughout the day coincident with meal times, and fail to show the drop-response to a meal [30,31]. The low pre-meal ghrelin in the TE constitutional type may be associated with their overweight tendencies reported in previous studies. However, the TE subjects in our study were not actually obese, so the low pre-meal ghrelin is not wholly explained by overweight status, indicating that the TE constitutional trait in itself could contribute as an independent factor to this particular ghrelin pattern.

In Sasang constitutional typology, it is generally understood that the SE type has smaller appetite and poor eating capacity, contrasting with the TE type who displays larger appetite and better eating capacity. Our study showed normal gut hormone profiles in the SE type, whereas the other types displayed several abnormal patterns, despite being normal-weight, and this may hint at disruptions in their appetite-controlling pathways that can eventually lead to aberrant eating behaviors or overweight/obesity. Especially, the strong association between obesity and the TE constitutional type is supported by many studies [32-34].

The limitations of our study are as follows: First, this was a pilot study, and the sample size was not large enough to confer great statistical power. Second, the ELISA method was used rather than radioimmunoassay, lowering the overall accuracy. Third, subjects were included only if both specialists gave identical constitutional typing, eliminating a large group (n = 14 of 49) from the initial applicants. This may have led to selection bias, and yet it was the most reasonable method possible (while maintaining maximal accuracy) considering the difficulties of including the comprehensive process of routine constitutional typing in this small-scale study. These problems should be addressed in future, larger-scale studies.

Though no final conclusions can be drawn from this pilot study, it is significant in that it approaches the association between gut hormone, appetite, and eating behavior through a different perspective, from the angle of individually diversified constitutional make-up, and also in that it is the first research on gut hormone profiles in different Sasang constitutional types.

## Conclusions

Upon examining the gut hormone profiles in healthy, normal-weight male subjects, meaningful gut hormone patterns specific to each Sasang constitutional type were discovered. Though the statistical power of this pilot study was weak, it nevertheless opens up new possibilities in future investigations on individual variability in appetite.

## Abbreviations

SCM: Sasang constitutional medicine; TY type: Taeyangin; SY type: Soyangin; TE type: Taeeumin; SE type: Soeumin; PYY: Peptide YY; GLP-1: Glucagon-like peptide 1; BULIT-R: Bulimia Test Revised; KEAT-26: Korean Version of Eating Attitude Test-26; GSRS: Gastrointestinal Symptom Rating Scale; DEBQ: Dutch Eating Behavior Questionnaire.

## Competing interests

The authors declare that they have no competing interests.

## Author’s contributions

The authors have contributed in the following ways: JL in the conception, designing, statistical analysis, and writing of the article; JL in the critical revision of the article; HS in data collection, KK in analysis and interpretation; EL in the critical revision of the article; BK in the critical revision of the article; and HJ in giving the final approval of the article. All authors read and approved the final manuscript.

## Pre-publication history

The pre-publication history for this paper can be accessed here:

http://www.biomedcentral.com/1472-6882/12/122/prepub

## References

[B1] Karra E Batterham RL The role of gut hormones in the regulation of body weight and energy homeostasis Mol Cell Endocrinol 2010 316 2 120 128 10.1016/j.mce.2009.06.010 19563862

[B2] Neary MT Batterham RL Gut hormones: implications for the treatment of obesity Pharmacol Ther 2009 124 1 44 56 10.1016/j.pharmthera.2009.06.005 19560488

[B3] Hameed S Dhillo WS Bloom SR Gut hormones and appetite control Oral Dis 2009 15 1 18 26 10.1111/j.1601-0825.2008.01492.x 18939959

[B4] Depoortere I De Clercq P Svoboda M Bare L Peeters TL Identification of motilin mRNA in the brain of man and rabbit. Conservation of polymorphism of the motilin gene across species Peptides 1997 18 10 1497 1503 10.1016/S0196-9781(97)00227-1 9437708

[B5] Park S Rew J Lee S Ki H Lee K Cheo J Kim H Noh D Joo Y Kim H Association of CCK(1) Receptor Gene Polymorphisms and Irritable Bowel Syndrome in Korean J Neurogastroenterol Motil 2010 16 1 71 76 10.5056/jnm.2010.16.1.71 20535329 PMC2879831

[B6] Lavebratt C Alpman A Persson B Arner P Hoffstedt J Common neuropeptide Y2 receptor gene variant is protective against obesity among Swedish men Int J Obes 2006 30 3 453 459 10.1038/sj.ijo.0803188 16331299

[B7] Song I Koh B Lee E Kim K Kim D Park S Sasang Constitutional Medicine 2004 Seoul: Jipmoondang

[B8] Kim JY Pham DD Sasang constitutional medicine as a holistic tailored medicine Evid Based Complement Alternat Med 2009 6 Suppl 1 11 19 19745007 10.1093/ecam/nep100 PMC2741623

[B9] Lee J Jung Y Yoo J Lee E Koh B Perspective of the human body in sasang constitutional medicine Evid-based Complement Altern Med 2009 6 Suppl 1 31 41 10.1093/ecam/nep086 PMC2741629 19745009

[B10] Kim E Kim J A clinical study on the Sasang constitution and obesity J Sasang Constitut med 2004 16 1 100 111

[B11] Lee K Seok J Kim S Kim Y Lee S Lee E Kim D Koh B A case - control study on risk factors of obese patients of each Sasang constitution J Sasang Constitut med 2007 19 2 94 112

[B12] Choi K Yoo J Lee E Koh B Lee J Sasang constitutional types can act as a risk factor for insulin resistance Diabetes Res Clin Pract 2011 91 3 e57 e60 10.1016/j.diabres.2010.11.017 21146241

[B13] Yin CS Park HJ Chung JH Lee HJ Lee BC Genome-wide association study of the four-constitution medicine J Altern Complement Med 2009 15 12 1327 1333 10.1089/acm.2009.0205 19954339

[B14] Kim M Yoo J Koh S Park J Prevalence of hypertension and risk factors according to Sasang constitution J Sasang Constitut med 2009 21 1 150 164

[B15] Lee T Hwang M Lee S Choe B Song I A study on the prevalence and risk factors of the metabolic syndrome according to Sasang constitution J Korean Oriental Med 2006 27 2 14 22

[B16] Hwang M Lee S Choe B Song I Koh B The research on the Sasang constitutional characteristics of stroke inpatients J Sasang Constitut med 2005 17 1 103 119

[B17] Hwang M Lee T Lee S Song I Choe B Koh B The case–control study of ischemic stroke according to Sasang constitution J Korean Oriental Med 2006 27 1 118 129

[B18] Park G Lee J Lee S Lee E Kim D Song I Koh B The study on the actual nutrient intake based on Sasang constitution J Sasang Constitut med 2007 19 3 188 205

[B19] Welch G Thompson L Hall A The BULIT-R: its reliability and clinical validity as a screening tool for DSM-III-R bulimia nervosa in a female tertiary education population Int J Eat Disord 1993 14 1 95 105 10.1002/1098-108X(199307)14:1<95::AID-EAT2260140113>3.0.CO;2-Z 8339105

[B20] Garner DM Garfinkel PE The eating attitudes test: an index of the symptoms of anorexia nervosa Psychol Med 1979 9 2 273 279 10.1017/S0033291700030762 472072

[B21] Svedlund J Sjodin I Dotevall G GSRS–a clinical rating scale for gastrointestinal symptoms in patients with irritable bowel syndrome and peptic ulcer disease Dig Dis Sci 1988 33 2 129 134 10.1007/BF01535722 3123181

[B22] Kim H Lee I Kim J A study of the reliability and validity of the korean version of the eating behavior questionnaire Kor J Clin Psychol 1996 15 1 141 150

[B23] Wardle J Eating style: a validation study of the dutch eating behaviour questionnaire in normal subjects and women with eating disorders J Psychosomat Res 1987 31 2 161 169 10.1016/0022-3999(87)90072-9 3473234

[B24] Adrian TE Ferri GL Bacarese-Hamilton AJ Fuessl HS Polak JM Bloom SR Human distribution and release of a putative new gut hormone, peptide YY Gastroenterology 1985 89 5 1070 1077 3840109 10.1016/0016-5085(85)90211-2

[B25] le Roux CW Batterham RL Aylwin SJ Patterson M Borg CM Wynne KJ Kent A Vincent RP Gardiner J Ghatei MA Attenuated peptide YY release in obese subjects is associated with reduced satiety Endocrinology 2006 147 1 3 8 10.1016/j.ygcen.2005.12.009 16166213

[B26] Turton MD O'Shea D Gunn I Beak SA Edwards CM Meeran K Choi SJ Taylor GM Heath MM Lambert PD A role for glucagon-like peptide-1 in the central regulation of feeding Nature 1996 379 6560 69 72 10.1038/379069a0 8538742

[B27] Orskov C Holst JJ Nielsen OV Effect of truncated glucagon-like peptide-1 [proglucagon-(78–107) amide] on endocrine secretion from pig pancreas, antrum, and nonantral stomach Endocrinology 1988 123 4 2009 2013 10.1210/endo-123-4-2009 2901341

[B28] Young AA Gedulin BR Rink TJ Dose-responses for the slowing of gastric emptying in a rodent model by glucagon-like peptide (7–36) NH2, amylin, cholecystokinin, and other possible regulators of nutrient uptake Metab Clin Exp 1996 45 1 1 3 10.1016/S0026-0495(96)90192-4 8544764

[B29] Cummings DE Purnell JQ Frayo RS Schmidova K Wisse BE Weigle DS A preprandial rise in plasma ghrelin levels suggests a role in meal initiation in humans Diabetes 2001 50 8 1714 1719 10.2337/diabetes.50.8.1714 11473029

[B30] le Roux CW Patterson M Vincent RP Hunt C Ghatei MA Bloom SR Postprandial plasma ghrelin is suppressed proportional to meal calorie content in normal-weight but not obese subjects J Clin Endocrinol Metab 2005 90 2 1068 1071 15522935 10.1210/jc.2004-1216

[B31] Shiiya T Nakazato M Mizuta M Date Y Mondal MS Tanaka M Nozoe S Hosoda H Kangawa K Matsukura S Plasma ghrelin levels in lean and obese humans and the effect of glucose on ghrelin secretion J Clin Endocrinol Metab 2002 87 1 240 244 10.1210/jc.87.1.240 11788653

[B32] Um JY Kim HM Mun SW Song YS Hong SH Interleukin-1 receptor antagonist gene polymorphism and traditional classification in obese women Int J Neurosci 2006 116 1 39 53 10.1080/00207450690962334 16318998

[B33] Song J Jeong H Kim S Son M Na H Song Y Hong S Kim H Um J Interleukin-1alpha polymorphism -889C/T related to obesity in Korean Taeumin women Am J Chin Med 2008 36 1 71 80 10.1142/S0192415X0800559X 18306451

[B34] Lee J Kwon Y Hong S Jeong H Kim H Um J Interleukin-1 beta gene polymorphism and traditional constitution in obese women Int J Neurosci 2008 118 6 793 805 10.1080/00207450701242883 18465425

